# Validation of an ambulatory cough detection and counting application using voluntary cough under different conditions

**DOI:** 10.1186/1745-9974-6-3

**Published:** 2010-05-27

**Authors:** Eldad Vizel, Mordechai Yigla, Yulia Goryachev, Eyal Dekel, Vered Felis, Hanna Levi, Isaac Kroin, Simon Godfrey, Noam Gavriely

**Affiliations:** 1 KarmelSonix Ltd., Haifa, Israel; 2 Pulmonary Section, Rambam Medical Center, Rappaport Faculty of Medicine, Technion - Israel Institute of Technology, Israel

## Abstract

**Background:**

While cough is an important defence mechanism of the respiratory system, its chronic presence is bothersome and may indicate the presence of a serious disease. We hereby describe the validation process of a novel cough detection and counting technology (PulmoTrack-CC™, KarmelSonix, Haifa, Israel).

**Methods:**

Tracheal and chest wall sounds, ambient sounds and chest motion were digitally recorded, using the PulmoTrack^® ^hardware, from healthy volunteers coughing voluntarily while **(a) **laying supine, **(b) **sitting, **(c) **sitting with strong ambient noise, **(d) **walking, and **(e) **climbing stairs, a total of 25 minutes per subject. The cough monitoring algorithm was applied to the recorded data to detect and count coughs.

The detection algorithm first searches for cough 'candidates' by identifying loud sounds with a cough pattern, followed by a secondary verification process based on detection of specific characteristics of cough. The recorded data were independently and blindly evaluated by trained experts who listened to the sounds and visually reviewed them on a sonogram display.

The validation process was based on two methods: **(i) **Referring to an expert consensus as gold standard, and comparing *each *cough detected by the algorithm to the expert marking, we marked True and False, positive and negative detections.These values were used to evaluate the specificity and sensitivity of the cough monitoring system. **(ii) **Counting the number of coughs in longer segments (t = 60 sec, n = 300) and plotting the cough count vs. the corresponding experts' count whereby the linear regression equation, the regression coefficient (*R*^2^) and the joint-distribution density Bland-Altman plots could be determined.

**Results:**

Data were recorded from 12 volunteers undergoing the complete protocol. The overall Specificity for cough events was 94% and the Sensitivity was 96%, with similar values found for all conditions, except for the stair climbing stage where the Specificity was 87% with Sensitivity of 97%. The regression equation between the PulmoTrack-CC™ cough event counts and the Experts' determination was with *R*^*2 *^of 0.94.

**Discussion:**

This validation scheme provides an objective and quantitative assessment method of a cough counting algorithm in a range of realistic situations that simulate ambulatory monitoring of cough. The ability to detect voluntary coughs under acoustically challenging ambient conditions may represent a useful step towards a clinically applicable automatic cough detector.

## Background

Cough is an important defence mechanism that helps clear secretions and air-bourn particles from the central airways [1]. A cough is a three-component respiratory maneuver starting with (i) an inspiration, followed by (ii) generation of an expiratory effort against the closed glottis and finally by (iii) rapid release of the intra-thoracic pressure resulting in expulsive expiratory flow [2,3]. When a single inspiration is followed by several expulsions or cough components it is called a multi-component cough [4]. The rapid expiratory flow of each cough component is associated with high air velocities that apply substantial inward Bernoulli forces on the tracheal walls and pull them inwards to a partial collapse. The cross section of the narrowed trachea further collapses and the flow velocity increases even more creating large shear forces between the moving air and the tracheal walls. It is these forces that carry with them the particles and excess secretions that lie on top of the mucosal lining of the airway [5,6].

While the cough reflex is essential in protecting the lung from foreign materials and infection, its excessive or chronic presence is both bothersome and potentially indicative of an on-going pathological process [7,8]. In particular, the situation where irritation of the cough receptors in the tracheal wall by the shear forces of one cough stimulate the generation of subsequent coughs creates an unending cycle that is sometimes hard to stop.

The assessment of coughing is currently subjective and based on the symptoms qualitative description as expressed by the patient or a parent. Quantitative and objective methods for cough assessment are not available beyond the investigative laboratory and are unique to the specific investigator (discussed for example in [9]). In addition to the clinical use, there is certain need for objective cough assessment for evaluation of newly developed cough medications. A recent position paper by the ERS Committee on cough clearly outlined the need for such objective cough assessment technology [7].

Patients with respiratory infection, asthma, COPD, Chronic Bronchitis, CF, lung fibrosis, GERD, Upper-Airway Syndrome, and others suffer from a multitude of pathologies of airways and are often inflicted with debilitating chronic cough.

Treatment of cough in these patients consists of many types of expectorants, cough suppressors, secretion modifiers, inhaled bronchodilators etc. In addition, chest physical therapy (PT) is often prescribed as part of the treatment regime. Assessing the efficacy of such treatment modalities is qualitative at best, particularly in young children and during the night.

The primary objective of this study was to develop a practical evaluation scheme to assess the efficacy and validity of an automatic cough counting application.

## Methods

### Setup

The study population consisted of 12 healthy adult volunteers, (6 Male) age 38 ± 13 (range 24-57) who signed informed consent to participate in the study. The study was approved by the Ethics Committee of Rambam Medical Center, Haifa, Israel and was conducted in an ambulatory setting outside the hospital. All subjects signed an informed consent form prior to participation in the study.

Table 1 outlines the study design. Recordings were made while the subject was **(a) **laying supine, **(b) **sitting, **(c) **sitting with strong ambient noise, **(d) **walking, and **(e) **climbing up and down stairs. Each phase lasted 5 minutes (25 minutes in total) in which the subject first did not cough for 2 minutes, then voluntarily coughed for 2 minutes then performed voluntary coughs of graded intensity, throat clears andtalking (counting from one to ten) for 1 minute. The mobile recordings (phases d+e) for research were performed while the subject was carrying the battery-operated recording system (PulmoTrack 2010™) inside a backpack, but for clinical use a small mobile system is now available similar in size to a cardiac Holter monitor. Two Phonopneumography (PPG) piezoelectric sensors were attached to the anterior neck (over the trachea) and chest and a pneumogram belt was placed at the xyphoid level.

**Table 1:** Study Design

Study phase	Duration	Activities
Supine	5 minutes	2 minutes with no cough 1 minute with 2-5 coughs events 1 minute with 5-8 coughs events 1 minute with weak and strong coughs followed by 3 throat clearings and speech from the patient.

Sitting	5 minutes	As above

Sitting, while a recording of music, coughs and speech is played in high volume in the background	5 minutes	As above

Walking	5 minutes	As above

Climbing up and down stairs	5 minutes	As above

### Analysis of data for cough detection and counting

The data recorded by the PulmoTrack^® ^were analyzed to calculate the following parameters:

✓ The timing of each cough event (i.e. a single- or multi- expulsion cough in a single breath, also known as cough "epochs" or "bouts" [10,11]) and each cough expulsion component.

✓ The cough event and component count per minute.

The cough time and the cough count were calculated using a cough detector algorithm which automatically detects coughs using the inputs from the PulmoTrack^® ^channels, recorded both from the patient and the ambient environment. It uses a two step top-down analysis algorithm. In the first step cough "candidates" are identified based on energy characteristics and cough amplitude pattern previously established from voluntary and spontaneous coughs. In the second step the "candidates" are verified based on their fit to a cough pattern in both the time and frequency domains. The burst time of each detected cough is recorded by the algorithm. The cough count per minute is the total number of coughs detected by the algorithm in that minute of recording.

The algorithm output was evaluated using the following parameters:

1. The cough-counting by the cough detector was compared to the evaluation by a consensus of two experts who were trained to detect coughs by listening to the recordings. The experts used a digital audio processing program (Adobe Audition 2.0) to mark the beginning and end of each cough event and explosive component.

2. The match between the cough count by the algorithm and the experts' determination was evaluated by determining if a detection by the cough detector algorithm was true positive (*TP*), true negative (*TN*), false positive (*FP*), or false negative (*FN*).

3. The algorithm performance was compared to a consensus of the expert analysis. The database included 300 minutes (12 patients, 25 minutes each) and was analyzed independently by 2 experts. Only cough expulsion components that were agreed by both experts were considered in. The algorithm was not 'punished' for missing components that were detected by only one expert (*FN*). Similarly, the algorithm was not 'credited' with True-Positive detection for components that were detected by only one expert.

4. To determine true negative (*TN*), we examined the detection results in randomly selected 1-second long segments that did not contain coughs. These segments contained quiet recordings as well as periods of talking by the subject and/or ambient noises.

5. The sensitivity (*SENS*), specificity (*SPEC*) and positive predictive value (*PPV*) were calculated as: *SENS *= *TP*/(*TP*+*FN*); *SPEC *= *TN*/(*TN*+*FP*); and *PPV *= *TP*/(*TP*+*FP*).

6. To facilitate comparison to accuracy calculations by Matos and Birring et al [12] we calculated an alternative Specificity - "*Birring SPEC*", as function of:

a. Number of Cough Candidates that were rejected by the algorithm, and determined as 'not cough' by expert analysis (True Rejected Candidates - TRC).

b. Number of Cough Candidates that were accepted by the algorithm, and determined as 'not cough' by expert analysis (False Accepted Candidates - FAC).

c. .

7. To calculate cough count accuracy we correlated the number of cough events and the number of cough explosive components detected by the algorithm in each 1 minute segment with the corresponding count by the experts. The linear regression equation and coefficient were calculated, and a Bland-Altman plot was calculated, with 95% limits of agreement.

8. We also calculated and correlated the number of "cough seconds" [13] - The number of seconds per minute which contained any number of cough components - detected by the algorithm and by the experts, using linear regression.

## Results

All of the 12 subjects completed the entire protocol with a total of 300 minutes of recordings. The entire data base was included in the analysis except for throat clearing which the current algorithm was not designed to detect. The overall *SENS *for detection of cough events for the entire database was 0.96 with *SPEC *of 0.94 and *PPV *of 0.90. Table 2 shows the *SENS*, *SPEC*, and *PPV *of cough events detection for the individual study phases.

**Table 2:** Detection parameters for Cough Events

Phase	*SENS*	*SPEC*	*PPV*
Supine	0.96	0.98	0.97

Seated	0.96	0.96	0.93

Seated + noise	0.95	0.93	0.90

Walking	0.95	0.94	0.90

Climbing stairs	0.97	0.87*	0.79*

Overall	0.96	0.94	0.90

*indicates outlier specificity value

Table 3 shows the accuracy values for detection of individual explosive components. The overall "Birring Specificity" (as explained in the *Methods *above) is 0.98, with details regarding each study phase shown in Table 3.

**Table 3:** Detection parameters for Explosive Components

Phase	*SENS*	*SPEC*	*PPV*	*"Birring Specificity"*
Supine	0.90	0.98	0.98	0.99

Seated	0.90	0.96	0.96	0.99

Seated + noise	0.86*	0.93	0.93	0.98

Walking	0.91	0.94	0.93	0.99

Climbing stairs	0.92	0.87*	0.85*	0.97

Overall	0.90	0.94	0.93	0.98

*indicates outlier specificity values

Table 4 shows the accuracy values for the "cough-seconds" detection.

**Table 4:** Detection parameters for Cough Seconds

Phase	*SENS*	*SPEC*	*PPV*
Supine	0.98	0.99	0.98

Seated	0.98	0.97	0.96

Seated + noise	0.98	0.95	0.94

Walking	0.98	0.96	0.95

Climbing stairs	0.98	0.89*	0.86*

Overall	0.98	0.95	0.94

**Figure 1:**
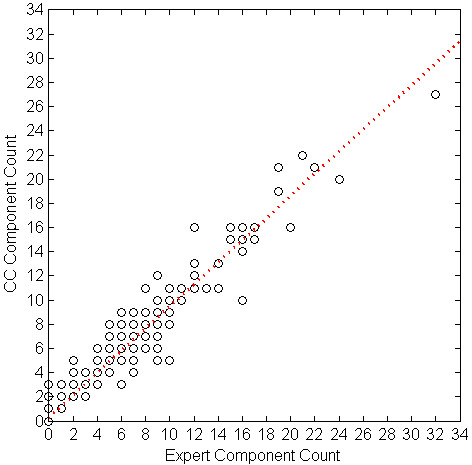
**Cough Counter vs. Expert and Linear Regression of Cough Component Count**. Markers indicate the algorithm (CC) and expert consensus count of Cough Components per Minute. Dashed line illustrates the slope and intercept of the linear regression.

**Figure 2:**
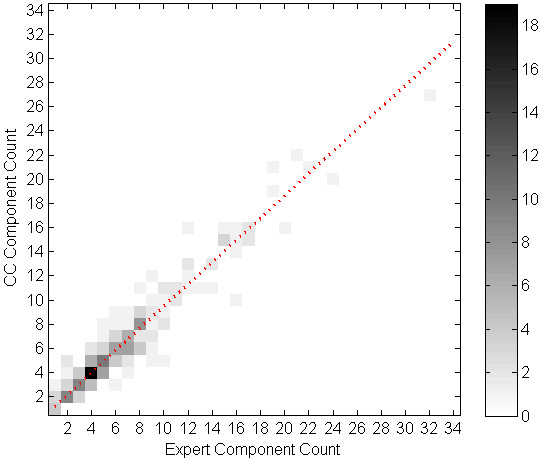
**Joint Distribution and Linear Regression of Cough Component Count: Greyscale indicates the number of examined minutes in each bin**. Dashed line illustrates the slope and intercept of the linear regression. Additional 99 minutes are in the [0,0] bin.

**Figure 3:**
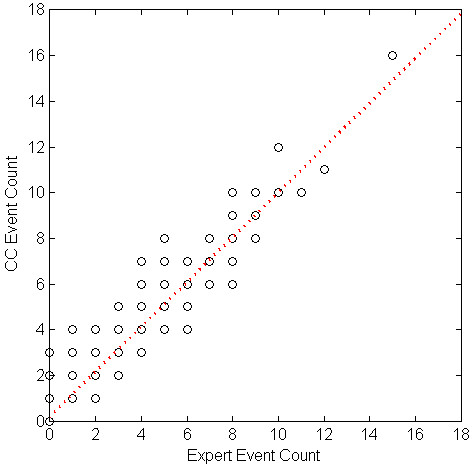
**Cough Counter vs. Expert and Linear Regression of Cough Event Count: Markers indicate the algorithm (CC) and expert consensus count of Cough Events per Minute**. Dashed line illustrates the slope and intercept of the linear regression.

**Figure 4:**
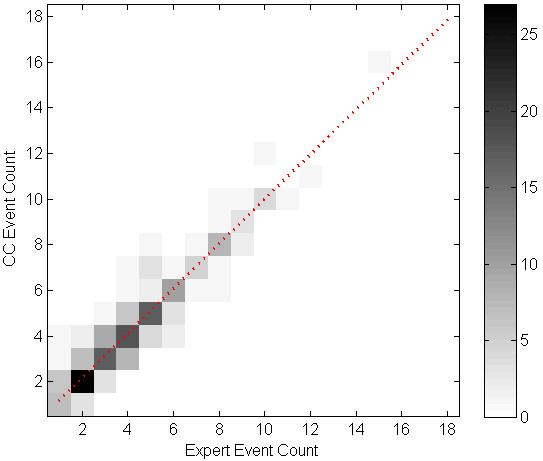
**Joint Distribution and Linear Regression of Cough Event Count: Greyscale indicates the number of examined minutes in each bin**. Dashed line illustrates the slope and intercept of the linear regression. Additional 99 minutes are in the [0,0] bin.

**Figure 5:**
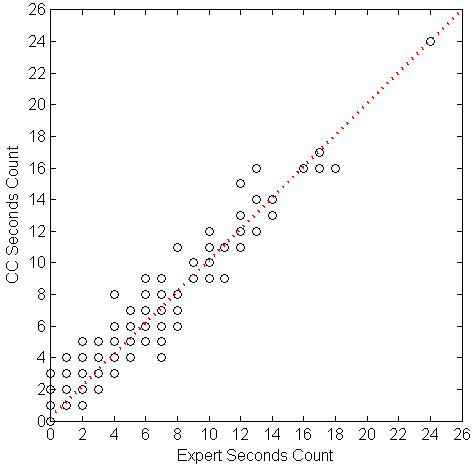
**Cough Counter vs. Expert and Linear Regression of Cough Second Count: Markers indicate the algorithm (CC) and expert consensus count of Cough Seconds per Minute**. Dashed line illustrates the slope and intercept of the linear regression.

**Figure 6:**
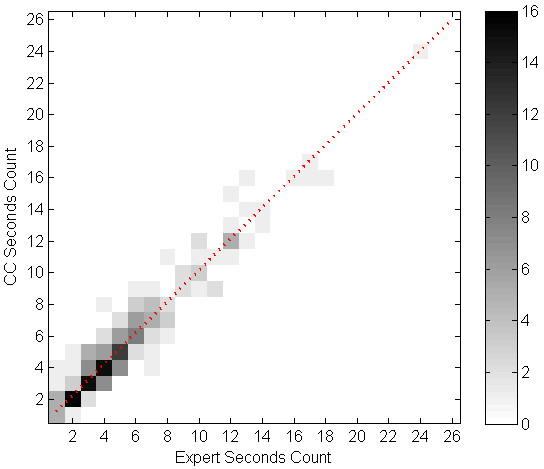
**Joint Distribution and Linear Regression of Cough Seconds Count: Greyscale indicates the number of examined minutes in each bin**. Dashed line illustrates the slope and intercept of the linear regression. Additional 99 minutes are in the [0,0] bin.

We evaluated the correlation of cough event count (per minute) between the algorithm and the experts' consensus using linear regression. Table 5 shows the parameters of the regression equation for the cough events, components, and seconds per study phase, and overall. All the intersect values of the regression equations were below 1. Table 6 shows SENS, SPEC, PPV and FP rate for each subject. Figures 1 through 6 illustrate the results of algorithm vs. expert analysis. A 'traditional' scatter plot is provided (fig. 1, 3, and 5), as well as a joint-distribution density graphs (fig. 2, 4, and 6).

**Table 5:** Regression parameters for Events, Components and Seconds

	Events		Components		Seconds	
**Phase**	**Slope**	***R*^*2*^**	**Slope**	***R*^*2*^**	**Slope**	***R*^*2*^**

Supine	0.97	0.97	0.93	0.98	0.95	0.95

Seated	1.00	0.97	0.89	0.97	1.00	0.98

Seated w. Noise	0.97	0.92	0.87	0.90	0.97	0.93

Walking	0.98	0.94	0.90	0.96	0.99	0.96

Climbing stairs	0.97	0.91	1.01	0.94	1.02	0.94

Overall	0.98	0.94	0.92	0.95	0.99	0.95

**Table 6:** SENS, SPEC, PPV and FP rate of each patient

Patient	Cough Components			Cough Events			Seconds Analysis			FP/Minute
	
	Sensitivity	Specificity	PPV	Sensitivity	Specificity	PPV	Sensitivity	Specificity	PPV	
1	0.94	0.95	0.92	0.95	0.95	0.9	0.98	0.95	0.91	0.02

2	0.97	0.92	0.89	0.98	0.92	0.86	1	0.95	0.92	0.02

3	0.93	0.96	0.93	0.93	0.96	0.89	0.94	0.97	0.94	0.01

4	0.9	0.9	0.85	0.94	0.9	0.73	0.98	0.92	0.84	0.03

5	0.83	0.92	0.92	0.89	0.92	0.89	0.94	0.95	0.95	0.02

6	0.95	1	1	0.98	1	1	1	1	1	0

7	0.98	0.95	0.89	1	0.95	0.86	1	0.95	0.87	0.02

8	0.82	0.94	0.94	0.92	0.94	0.91	0.97	0.96	0.95	0.02

9	0.9	0.95	0.95	0.96	0.95	0.94	0.98	0.96	0.95	0.02

10	0.92	0.87	0.92	0.97	0.87	0.85	0.98	0.89	0.91	0.05

11	0.9	0.96	0.97	0.98	0.96	0.95	0.99	0.97	0.97	0.01

12	0.8	0.89	0.85	0.95	0.89	0.81	1	0.93	0.89	0.04

Average	0.90	0.93	0.92	0.95	0.93	0.88	0.98	0.95	0.93	0.02

SD	0.06	0.04	0.04	0.03	0.04	0.07	0.02	0.03	0.04	0.01

The joint-distribution graphs illustrate how many occurrences were found for each combination of expert and algorithm counts per minute. Dark grey indicates a high number of occurrences, while light shades indicate a low number. For example in fig. 4, the (2,2) bin is dark-grey, indicating over 25 minutes where the expert counted 2 cough events, and the algorithm counted 2 events as well, for the same evaluated minutes.

**Figure 7:**
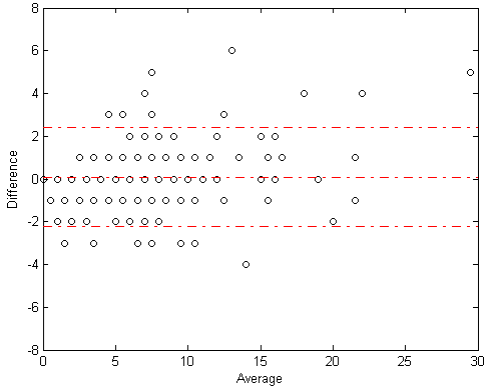
**Bland-Altman plot for cough components**. The dashed lines represent the mean difference ± 1.96 SD. 282 out of 300 minutes (94%) of the measurement are within these limits. Also note that the algorithm to expert difference was not dependent on the overall number of coughs in each segment.

**Figure 8:**
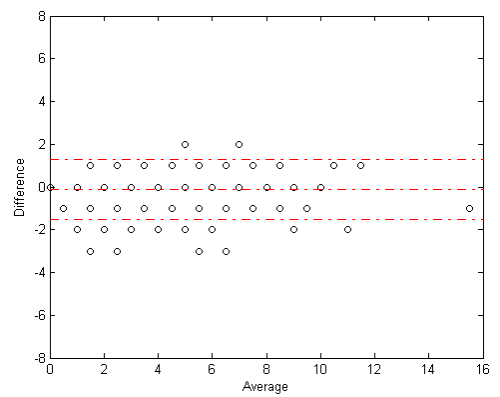
**Bland-Altman plot for cough events**. The dashed lines represent the mean difference ± 1.96 SD. 282 out of 300 (94%) minutes of the measurement are within these limits. Also note that the algorithm to expert difference was not dependent on the overall number of coughs in each segment.

**Figure 9:**
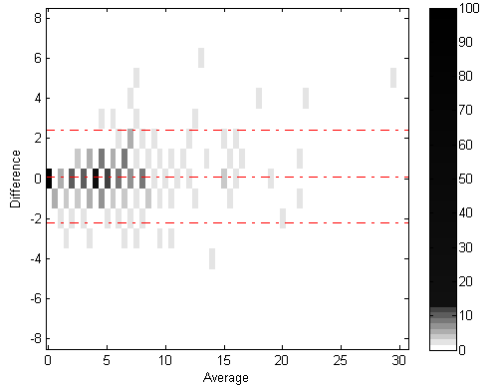
**Bland-Altman Joint Distribution density for cough components**. The dashed lines represent the mean difference ± 1.96 SD.

**Figure 10:**
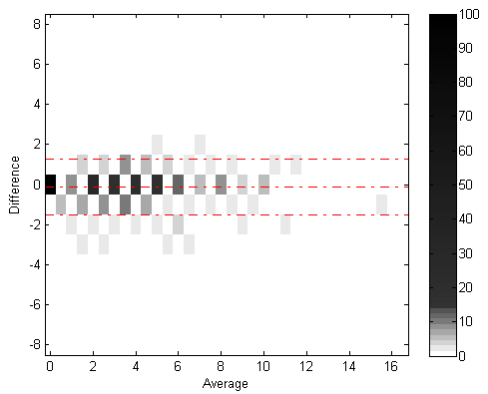
**Bland-Altman Joint Distribution for cough events**. The dashed lines represent the mean difference ± 1.96 SD.

Figures 7 and 8 show Bland-Altman plots for correlation between algorithm and expert cough components and events count. Figures 9 and 10 show the joint-distribution density Bland-Altman plots.

All values of *SPEC*, *SENS*, *PPV*, Slope and *R*^2 ^except when climbing stairs were above 0.9 and close to unity. The accuracy of detecting at least one component in a cough event was statistically greater than that of detecting an individual explosive component with event sensitivity of 96% and individual explosive component sensitivity being 91%. We did not identify a systematic type of repeating false positive or negative detection, except false positive detection of few bursts of laughter.

## Discussion

We describe a validation method and results for determining the accuracy of a novel cough detection technology. A database was collected from normal healthy volunteers who voluntarily coughed according to a structured protocol during sedentary and ambulatory conditions. Additionally, we included a recording phase where significant ambient noises were imposed. These challenging conditions were used to evaluate the accuracy of the algorithm under realistic or even challenging conditions.

The entire database was then evaluated by trained experts who listened to all the recordings to identify the coughs. The experts were blinded to the PulmoTrack-CC™ results. The experts used a combined time/frequency display to mark the exact beginning and end of each cough component. They also marked the number of cough components per each cough event. Audio recordings were previously established as adequate for locating and counting cough components and events [14].

The overall sensitivity of the algorithm in detecting cough events and cough seconds was very high (0.96-0.98) with a somewhat lower sensitivity (0.90) in detecting individual components. It has been shown in a recent study that cough epochs (events) correlated slightly less strongly than cough components with the Leicester Cough Questionnaire (LCQ) [13]. The specificity was also very high (0.94-0.95) except when climbing up and down the stairs. In addition to the Event and Component detection parameters, we calculated the parameters based on "cough seconds" to facilitate comparison to published studies that used this approach to determine the extent of coughing. Likewise, we calculated a parameter we suggest calling the "Birring Specificity" to facilitate comparison to the data published by Birring et al [12-15]. In general, the accuracy of the PulmoTrack-CC method for voluntary coughs matches or exceeds that of all other published cough detection methods applied to spontaneous coughs [12,14,15]. This is despite the fact that the validation database described herewith imposes deliberate challenging conditions on the detection algorithm. However, since the detected coughs were voluntary, further validation is needed in 24 hours ambulatory recordings of patients with respiratory diseases. Clearly, a study with spontaneous cough under natural ambulatory conditions is called for.

The PulmoTrack-CC™ uses data from the PPG Sensors with supporting information from the pneumograph belt and the ambient microphone. It should be noted that the algorithm is fully automatic with no need for pre-training of the algorithm with the individual patient. Additionally, there is no need for manual or operator intervention in the detection process. The processing of each 60 seconds of record requires less than 10 seconds, depending on the type of CPU processor.

## Conclusions

This study describes a database of voluntary coughs that was designed specifically to test and validate the accuracy of a new cough detector technology under various challenging conditions. We describe the method used to compare the results of the automated cough detector and the determination by experts using established acoustic technology. We suggest that this method could be used to evaluate cough detection systems in a standardized fashion under experimental and clinical conditions.

## Competing interests

Dr. Igla: none. All other authors are paid officers or employees of KarmelSonix Ltd and hold shares and/or stock options in the Company.

## Authors' contributions

EV developed the algorithms and participated in writing the manuscript. MY oversaw the medical aspects of the study, YG supervised the data acquisition and performed the data analysis, ED participated in editing the manuscript, VF collected the experimental data, HL prepared the experimental protocol and assisted in the statistical data analysis, IK developed the hardware, SG evaluated the statistical data analysis and its presentation, NG oversaw the project and the manuscript preparation. All authors took active part in the project, reviewed and contributed to the manuscript and take responsibility for its accuracy.
